# Supplementary material for: Bayesian inference of state feedback control parameters for fo perturbation responses in cerebellar ataxia
Source: PLoS Comput Biol. 2024 Oct 11;20(10):e1011986. doi: 10.1371/journal.pcbi.1011986 (PMC11498721; doi:10.1371/journal.pcbi.1011986)
Supplement: S1 Appendix — (PDF) [file pcbi.1011986.s001.pdf]

Supporting information

Observer Estimate of Feedback Noise

S1 Appendix To investigate the relationship between the feedback noise parameters and Kalman gain, two additional experiments were conducted. In the original model, the Kalman gain calculation is assumed to have perfect knowledge of the variance of noise in auditory and somatosensory feedback. In these additional experiments, the variance of noise added to the sensory feedback signals was distinguished from the observer's internal estimate of noise variance used to calculate Kalman gain. First, the simulation-based inference procedures were repeated with the observer's estimate of noise variance fixed to the inferred values of the control group. In other words, optimal fit values were inferred for all five parameters, including the sensory noise parameters, but the influence of the noise parameters on Kalman gain was ablated. Second, the simulation-based inference procedures were repeated with the condition that the noise parameters affected only the observer's estimate of sensory noise, while the actual noise added to the sensory signals in the plant was ablated. We can see in Fig 1 that ablating the observer's estimate only produces nearly the same error as ablating the feedback noise ratio parameter, while ablating the actual noise and not the observer's estimate has negligible impact on fit accuracy compared with the full model. We can therefore conclude that the main impact of the feedback noise ratio parameter is in its role in the calculation of Kalman gain.

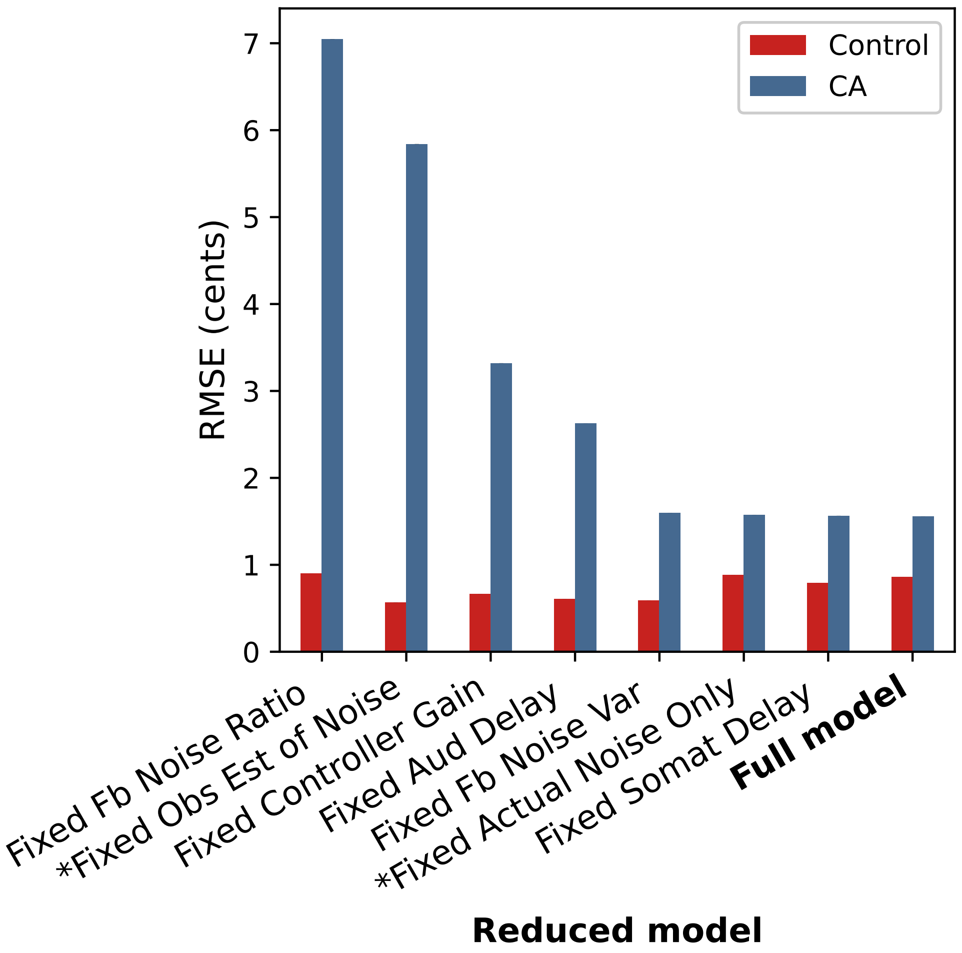

Fig 1. Noise parameters influence simulator output mainly through the calculation of Kalman gain. A large impact on fit accuracy is observed when the observer's estimate of sensory feedback noise is ablated, however, no loss of fit accuracy is observed when the actual sensory feedback noise is fixed and the observer's estimate of noise is optimized.
